# Immunization with Live Human Rhinovirus (HRV) 16 Induces Protection in Cotton Rats against HRV14 Infection

**DOI:** 10.3389/fmicb.2017.01646

**Published:** 2017-08-31

**Authors:** Mira C. Patel, Lioubov M. Pletneva, Marina S. Boukhvalova, Stefanie N. Vogel, Adriana E. Kajon, Jorge C. G. Blanco

**Affiliations:** ^1^Sigmovir Biosystems, Inc., Rockville MD, United States; ^2^University of Maryland School of Medicine, Baltimore MD, United States; ^3^Infectious Disease Program, Lovelace Respiratory Research Institute, Albuquerque NM, United States

**Keywords:** rhinoviruses, cross-protection, vaccines, cotton rat, asthma

## Abstract

Human rhinoviruses (HRVs) are the main cause of cold-like illnesses, and currently no vaccine or antiviral therapies against HRVs are available to prevent or mitigate HRV infection. There are more than 150 antigenically heterogeneous HRV serotypes, with ∼90 HRVs belonging to major group species A and B. Development of small animal models that are susceptible to infection with major group HRVs would be beneficial for vaccine research. Previously, we showed that the cotton rat (*Sigmodon hispidus*) is semi-permissive to HRV16 (major group, species HRV-A virus) infection, replicating in the upper and lower respiratory tracts with measurable pathology, mucus production, and expression of inflammatory mediators. Herein, we report that intranasal infection of cotton rats with HRV14 (major group, species HRV-B virus) results in isolation of infectious virus from the nose and lung. Similar to HRV16, intramuscular immunization with live HRV14 induces homologous protection that correlated with high levels of serum neutralizing antibodies. Vaccination and challenge experiments with HRV14 and HRV16 to evaluate the development of cross-protective immunity demonstrate that intramuscular immunization with live HRV16 significantly protects animals against HRV14 challenge. Determination of the immunological mechanisms involved in heterologous protection and further characterization of infection with other major HRV serotypes in the cotton rat could enhance the robustness of the model to define heterotypic relationships between this diverse group of viruses and thereby increase its potential for development of a multi-serotype HRV vaccine.

## Introduction

Human rhinoviruses (HRVs) are single-stranded, positive-sense RNA viruses of the family *Picornaviridae*, genus *Enterovirus*, and the most common cause of upper respiratory tract (URT) infection worldwide (Jacobs et al., 2013). HRVs are frequently detected in association with hospitalizations for acute respiratory illness in young children and the elderly and are also a frequent opportunistic pathogen of transplant recipients and immunocompromised patients (Kaiser et al., 2006; Milano et al., 2010; Iwane et al., 2011; Kraft et al., 2012). In addition, HRV infections have been associated with exacerbation episodes in asthmatic and chronic obstructive pulmonary disease (COPD) patients (Nicholson et al., 1993; Johnston et al., 1995; Rakes et al., 1999; Papi et al., 2006). Considering the high frequency of HRV infections in humans and the strong evidence supporting their roles as an inducer or effector of atopy or asthma, effective control of HRV infections through treatment and prevention would have significant public health benefit.

Nearly 60 years after their isolation, there are no approved antiviral therapies for the prevention or treatment of HRV infections (Jacobs et al., 2013). Currently, three genetically distinct groups, HRV-A, -B, and -C, have been characterized on the basis of genomic structure (Savolainen et al., 2002; Lau et al., 2007; Palmenberg et al., 2009; Simmonds et al., 2010; Bochkov et al., 2011). Seventy-five serotypes of HRV-A and 25 serotypes of HRV-B are antigenically distinct viruses based on cross-neutralization properties *in vitro* (Kapikian et al., 1967; Hamparian et al., 1987). Based on competition for cellular binding sites, two different groups of HRVs using non-identical receptors for cell attachment were defined. Approximately 10 minor group serotypes (1A, 1B, 2, 29, 30, 31, 44, 47, 49, and 62) use the low-density lipoprotein (LDL) as the receptor for cell entry (Hofer et al., 1994; Marlovits et al., 1998), while >90% of major group serotypes, including HRV16 (species HRV-A) and HRV14 (species HRV-B), utilize intercellular adhesion molecule 1 (ICAM-1) receptor (Greve et al., 1989). Some major group HRVs also use heparin sulfate as an additional receptor (Vlasak et al., 2005). The HRV-C group of viruses does not infect conventional cell lines used for virus propagation (i.e., HeLa or embryonic fibroblasts). Recently, Cadherin-related family member 3 was characterized as the receptor for the HRV-C and HRV-C15 was propagated using reverse genetics facilitating the isolation of HRV-C strains (Bochkov et al., 2011, 2015). In addition, HRV-C viruses have been shown to grow in sinus mucosal tissue or differentiated sinus epithelial cells (Bochkov et al., 2011; Ashraf et al., 2013). Efforts at vaccine development have been hindered because there are more than 150 HRV serotypes with extensive antigenic heterogeneity and broad circulation (Savolainen et al., 2002; Lau et al., 2007; Palmenberg et al., 2009; Simmonds et al., 2010; Bochkov et al., 2011).

An experimental animal model that is susceptible to different HRV serotypes would be pivotal to evaluate the degree of cross-protection *in vivo*, to delineate anti-HRV vaccine strategies, or to conduct preclinical studies of antiviral compounds. Since 2008, multiple studies have evaluated a mouse model for rhinovirus-induced disease and exacerbation of allergic airway inflammation (Bartlett et al., 2008; McLean et al., 2012; Glanville et al., 2013). Using minor group HRV1B serotype, Bartlett et al. (2008) demonstrated that infection in BALB/c mouse is localized in the lungs and induces strong airway and pulmonary inflammation and mucin production, albeit accompanied by low viral replication. In addition, a model for major group HRV16 serotype infection was also reported by using transgenic mice expressing a human/mouse ICAM-1 chimeric receptor (Bartlett et al., 2008). This model showed a strong inflammatory response to infection with low levels of viral replication similar to HRV1B in BALB/c mice. However, use of a transgenic mouse for infection with ∼90 major group viruses for *in vivo* cross-protection studies could become challenging. Therefore, development of an alternative small animal model that is susceptible to infection by major group HRVs would be a step forward to vaccine development.

Our group has recently showed that intranasal (i.n.) infection of cotton rats with HRV16 resulted in measurable isolation of infective virus in nose and lung tissues, lower respiratory tract pathology, mucus production, and expression of interferon (IFN)-activated genes without any genetic modification of either the host or the virus (Blanco et al., 2014). The cotton rat is an animal model frequently used to study infections by many respiratory viral pathogens that affect human health, including respiratory syncytial virus (RSV) (Boukhvalova and Blanco, 2013), influenza (Ottolini et al., 2005; Blanco et al., 2013), measles (Wyde et al., 1992; Pfeuffer et al., 2003), and the recently re-emerging Enterovirus-D68 (EV-D68) (Patel et al., 2016). We have previously demonstrated that intramuscular (i.m.) immunization of cotton rats with live HRV16 generates protective immunity that correlates with high levels of serum neutralizing antibodies (NA), which protect vaccinated animals as well as litters born to vaccinated females against HRV16 challenge. In addition, passive prophylactic treatment with hyperimmune anti-HRV16 serum protects naïve animals against i.n. challenge with HRV16 (Blanco et al., 2014). These results suggest that the cotton rat could become a useful model for testing vaccines and prophylactic therapies against major group of HRV infection.

In the present study, we have extended the capabilities of this model by reporting that i.n. infection of cotton rats with another major group species HRV-B rhinovirus, HRV14, also results in isolation of infective virus from nose and lung tissues. Importantly for vaccine purposes, we report an *in vivo* cross-protective relationship between HRV16 and HRV14 that has not been previously described. These results are a step toward defining a new level of cross-neutralization relationships among HRVs, which can shed new insight for development of a multi-serotype HRV vaccine.

## Materials and Methods

### Animals

Four to six week old cotton rats were obtained from the inbred colony maintained at Sigmovir Biosystems, Inc. (SBI). Sentinel cotton rats in the colony were seronegative for rhinoviruses (HRV16, 14, 1A, 1B) by neutralization assay, and seronegative to other adventitious respiratory viruses (e.g., pneumonia virus of mice, rat parvovirus, rat coronavirus, Sendai virus) by ELISA. Animals were housed in large polycarbonate cages, and fed a diet of standard rodent chow and water *ad libitum*. All animal work presented in this paper was conducted in accordance with the recommendations in the Guide for the Care and Use of Laboratory Animals of the National Institutes of Health. The animal protocols were approved by the Institutional Animal Care and Use Committee (IACUC) of SBI (OLAW assurance #A4642-01). Cotton rats were infected i.n. or immunized i.m. with either live HRV14 (stock titer: 3.97 × 10^7^ PFU/ml) or HRV16 (stock titer: 2.1 × 10^8^ PFU/ml) or UV-inactivated HRV14 (UV-HRV14) or UV-HRV16 under isoflurane anesthesia by inoculation of 100 μl of virus preparation [10^6^–10^7^ PFUs] per rat as indicated in the figure legends. Serum samples were obtained by retro-orbital blood collection under isoflurane anesthesia. Animals were euthanized by carbon dioxide asphyxiation. All the infection work with animals or tissue culture cells was strictly carried out at standard biosafety level-2.

### Virus and Cells

Stocks of HRV14 (ATCC cat. # VR-284) and HRV16 (ATCC cat. # VR-283) were produced in HeLa Ohio (HeLa OH) cells, a generous gift of Dr. Dean Erdman (CDC, Atlanta, GA, United States). Cells were grown in Minimal Essential Medium containing Earle salts (EMEM), 10% fetal bovine serum (FBS), 1.5 g/L NaHCO_3_, L-glutamine and penicillin/streptomycin at 33°C and 5% CO_2_. Virus stocks were prepared by infection of HeLa OH cell monolayers at a multiplicity of infection of approximately 0.1 PFU/cell. Infected cells were maintained at 33°C, harvested when extensive cytopathic effect (CPE) was evident around 48–72 h post infection (p.i.) and frozen at -80°C. Cells were thawed and subjected to one additional freeze-thaw cycle and clarified by centrifugation at 3,000 rpm for 5 min. Supernatant was extracted with one volume of water-saturated chloroform for additional elimination of lipid moieties and frozen at -80°C (Feldman and Wang, 1961; Deutsch and Wassermann, 1965). Infectious titers of virus stocks were determined by plaque assay on monolayers of HeLa OH cells in 6-well plates as described below. Inactivated preparations of each virus were generated by exposing the virus stocks to ultraviolet irradiation for 20 min at 100 mJ/cm^2^ and verified by plaque assay.

### Virus Titration Assay

Tissue samples (left lung lobe and entire nose) were homogenized in 3 ml of homogenization buffer (EMEM, 2% FBS, 1.5 g/L NaHCO_3_, 25 mM HEPES, penicillin and streptomycin) at different time-points p.i. Infectious virus titers were determined by standard plaque assay and expressed as plaque forming units (PFU)/g of tissue. Briefly, 100 μl of undiluted or tenfold serial diluted tissue homogenates or virus stocks were plated in triplicate onto confluent monolayers of HeLa OH cells in 6-well plates. After 1 h adsorption with rocking every 10 min, monolayers were over-layered with 2 ml of 0.7% low melt agarose in EMEM containing 2% FBS. Following incubation for 3 days at 33°C, monolayers were fixed with buffered formaldehyde and stained with crystal violet.

### Neutralizing Antibody Titer Assay

NA titers were determined by either plaque-reduction assay (**Figure 2**) or by CPE-reduction assay (**Figure 3D**). For plaque-reduction assay, serial four-fold dilutions of serum were incubated for 1 h at 37°C with ∼50 PFU of HRV14. Virus incubated with PBS was used as a control. Neutralization mixes were plated in quadruplicate onto confluent HeLa OH cell monolayers in 24-well plates, incubated at 33°C for 1 h and over-layered with 0.7% low melt agarose in EMEM containing 2% FBS. Cells were incubated at 33°C and 5% CO_2_ for 3 days, fixed with 1% buffered formaldehyde, and stained with crystal violet for quantification of plaques. For CPE-reduction assay, H1-HeLa cells (ATCC, Manassas, VA, United States) were seeded in 96-well plates to attain 90–95% confluence in 48 h. Heat-inactivated cotton rat sera were two-fold serially diluted and mixed with 500 TCID_50_ of either HRV14 or HRV16 in an equal volume and incubated at 33°C for 2 h. One hundred μl of the serum-virus mixture was transferred onto H1-HeLa cell monolayers in 96-well plates in duplicate, plates were incubated at 33°C and 5% CO_2_ for 5 days, stained with crystal violet as described above, and wells were scored for the presence of absence of CPE. For each plate, a no-serum and no virus controls were included. The number of protected wells was determined for each sera and NA titer was assigned on the basis of highest dilution that rendered protection of monolayer. We also used 1,000 TCID_50_ as input virus and serum-virus neutralization at 37°C for 6 h (Katpally et al., 2009), and found similar results.

### RNA Isolation and qRT-PCR Analysis

RNA was isolated from the lung lingular lobe using the RNeasy kit (Qiagen Sciences). cDNA was prepared by SuperScript^®^ II Reverse transcriptase (Thermo Fisher Scientific). Each cDNA reaction was prepared from 1 μg of total RNA and diluted to 100 μl of the final volume. Three μl of cDNA was subsequently used for each PCR reaction. HRV14 specific qRT-PCR was developed using primers that target the VP1 region of HRV14. To quantify viral replication, cDNA for the negative strand viral RNA [(-) vRNA] (an indicator of active RNA transcription) was synthesized by priming with 5′-TTTGCTAGCTTTAGGACCTACTATATCTACCTGGCCTCAATCTCATCTGGTC-3′, primer designed with the strategy described before (tagged primer by adding a 32-mer-long sequence of the Grapevine virus A as a tag at the 5′-end of the respective primer) (Lim et al., 2013). The real-time PCR reaction was performed using internal nested forward primer 5′-TGCCTGTTCTTCCATCAGAC-3′ and reverse primer 5′-ATCAGGTTGAGATGCAGTGG-3′. The VP1 PCR amplicon generated using forward primer 5′-GGCCTCAATCTCATCTGGTC-3′ and reverse primer 5′-AGGGTAGTGCTCTGGGTGCT-3′, was gel-purified, sequenced and diluted to generate a copy number standard curve, which was used to quantify copies/μg of RNA of (-) vRNA.

### Lung Histopathology

Lungs (right lobe) were dissected and inflated with 10% neutral buffered formalin to their normal volume and immersed in formalin for fixation. Lungs were embedded in paraffin blocks, sectioned, and stained with hematoxylin and eosin (H&E). Four parameters of pulmonary inflammation were evaluated: peribronchiolitis, perivasculitis, interstitial pneumonia, and alveolitis. Slides were assessed subjectively and scored blindly on a 0–4-severity scale (absent, minimal, mild, moderate, and marked; (Blanco et al., 2014).

### IFN-γ ELISPOT

HRV14 or HRV16-specific cellular immune responses were assessed by cotton rat specific IFN-γ ELISPOT assay. 96-well multiscreen plates (Millipore, Danvers, MA, United States) were coated overnight at 4°C with 1 μg/well of capture antibody (goat anti-cotton rat IFN-γ; R&D Systems, Inc., Minneapolis, MN, United States). The plates were washed three times with PBS containing 0.05% Tween-20 (PBS-Tween), blocked with PBS containing 5% FBS at 37°C for 2 h, washed three times with PBS-Tween, and rinsed twice with RPMI 1640 containing 10% FBS (splenocyte culture medium). Cotton rat splenocytes (5 × 10^5^) in duplicate were incubated with controls (culture media as well as supernatant of uninfected HeLa OH cells), HRV14 (UV-HRV14; 1 × 10^5^ PFU/well), HRV16 (UV-HRV16; 1 × 10^5^ PFU/well) specific stimuli, or Concanavalin A (Con A; 2.5 μg/ml) in 100 μl reaction mixture volume. Following 18 h incubation at 37°C, the plates were washed 10 times with PBS-Tween. Subsequently, plates were incubated with 2 μg/ml of the conjugated detection antibody (biotinylated goat anti-cotton rat IFN-γ; R&D Systems, Inc.) at room temperature, followed by washing plates six times with PBS-Tween. After incubation for 2 h at room temperature with 1:500 dilution of streptavidin-horseradish peroxidase (R&D Systems, Inc.), plates were washed five times with PBS-Tween and once with PBS. Plates were developed with ready-to-use TMB substrate for ELISPOT (MABTECH, Cincinnati, OH, United States), and read using Autoimmun Diagnostika MultiSpot reader system (AID GmbH, Strassberg; Janetzki et al., 2015).

### Statistical Analysis

Viral titers, NA titers, and (-) vRNA were calculated as geometric means ± standard error of the mean (SEM) for all animals in a group at a given time p.i. The Student’s *t*-test was used to determine statistically significant differences between two groups, using an unpaired, two-tailed test with significance set at *p* < 0.05. For **Figures 3B,C**, all data differences between immunization groups were assessed by one-way ANOVA, and if significant (*p* < 0.05), individual differences were identified using Bonferroni *post hoc* test.

## Results

### HRV14 Infection in the Cotton Rats

**FIGURE 1:**
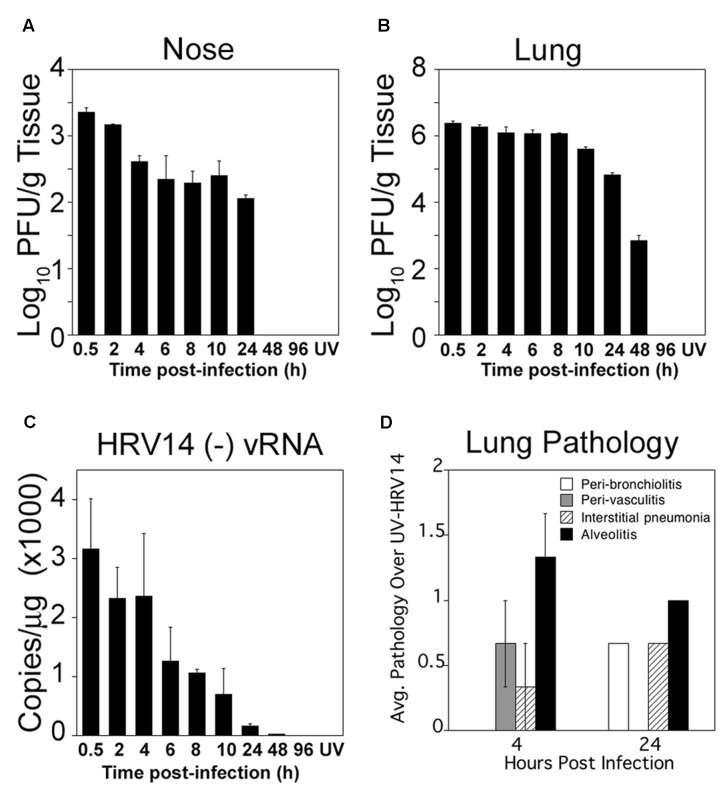
Cotton rat infection with HRV14. Cotton rats were i.n. infected with 10^6^ PFU/100 μl of HRV14. Infectious virus titers in nose **(A)**, and in lung **(B)** homogenates from infected animals at the indicated times (h) post-challenge. Groups of three animals were sacrificed at each time point. Results are representative of two independent experiments. Animals inoculated with UV-HRV14 and sacrificed at 4 h p.i. (UV) were shown as control. **(C)** Quantification of HRV14 (-) vRNA by qRT-PCR in lung tissue at the indicated times p.i. *n* = 3 per time point and results are representative of two independent experiments. **(D)** Histopathology scores obtained from lungs of HRV14 infected animals and euthanized at the indicated time p.i. The score of each parameter was plotted for HRV14-infected animals after subtracting the background lung inflammation induced by UV-HRV14 inoculation at each time analyzed. Data is representative of two independent experiments, *n* = 3/experiment.

Adult cotton rats were infected i.n. with 10^6^ PFU of HRV14. Groups of three animals were sacrificed at 0.5, 2, 4, 6, 8, 10, 24, 48, and 96 h p.i. Animals inoculated with UV-HRV14 (titer of 10^6^ PFU prior to inactivation) sacrificed 4 h post-inoculation were used as negative control for viral replication. Infectious HRV14 virus was recovered from the nose until 24 h p.i. and from the lung until 48 h p.i. (**Figures 1A,B**, respectively). No replicative virus was detected in animals infected and euthanized at 96 h p.i. or in UV-HRV14 inoculated control animals at 4 h post-inoculation. Higher virus titers were detected in the lungs (∼10^6^ PFU/g tissue) compared to the nose (∼10^3^ PFU/g tissue). A brief plateau of viral loads was detectable in the nose between 6 and 24 h and between 4 and 8 h p.i. in the lungs, followed by clearance of the virus. The viral load in the lung was also quantified by detecting the production of the replication intermediate, (-) vRNA by qRT-PCR (**Figure 1C**). The (-) vRNA strand was detected in the lungs of infected animals up to 24 h p.i., and decreased dramatically by 48 h p.i. (**Figure 1C**). Animals inoculated with UV-HRV14 and sacrificed at 4 h post-inoculation showed undetectable (-) vRNA (compare 4 h HRV14 with UV-HRV14 at 4 h in **Figure 1C**).

Next, we examined the extent of lung pathology followed by HRV14 infection at early (4 h p.i.), when virus load is higher, and late (24 h p.i.), when the virus load in the lung is strongly reduced (**Figure 1D**). HRV14 infection showed low levels of cellular infiltration, mostly around the bronchioles and within the alveolar spaces at 24 h p.i., that lead to mild but significant increase in pathology when compared to animals inoculated with UV-HRV14.

### Induction of Serum NA in Response to HRV14

**FIGURE 2:**
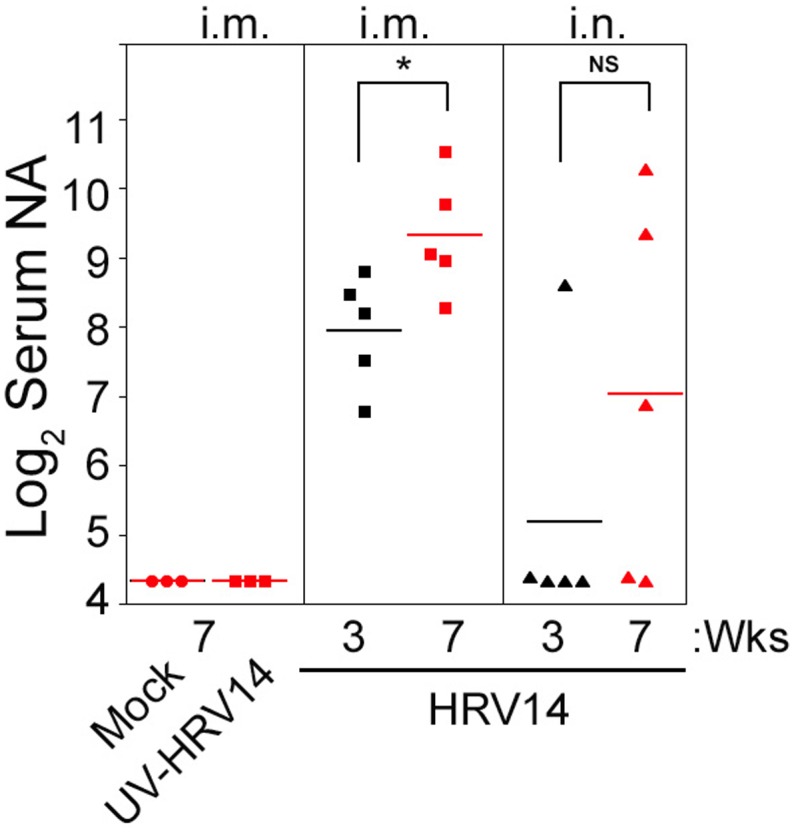
Profile of serum NA in i.m. immunized or i.n. infected cotton rats using HRV14. Female cotton rats were mock-immunized or i.m. immunized with 10^6^ PFU/100 μl of UV-HRV14 or HRV14 on day 0 and boosted 3 weeks after the first immunization. Another group of rats were i.n. infected 10^6^ PFU/100 μl of HRV14 on day 0 and re-infected at 3 weeks. Serum samples were obtained on the 3rd (black symbols) and 7th week (red symbols) after the first immunization or infection and homologous serum NA against HRV14 were analyzed. The sera were assayed in duplicate and NA titer is expressed as Log_2_ geometric mean ± SEM of the reciprocal dilution of serum samples that achieved 60% reduction of the total plaques. ^∗^*p* < 0.05 in Student *t-*test comparison between 3 weeks vs. 7 weeks i.m. immunized or i.n. infected groups. *n* = 3–5 per group.

We next investigated the effect of i.m. immunization with either live or UV-HRV14 or i.n. infection on the generation of serum NA against HRV14. Live HRV14 or UV-HRV14 were given i.m. at the dose of 10^6^ PFU at day 0 and boosted 3 weeks after the first immunization. Another group of cotton rats were infected i.n. with HRV14 at the dose of 10^6^ PFU on day 0 and re-infected after 3 weeks. Serum samples were collected from animals at 3 (before boosting or re-infection) and 7 weeks after the first immunization or infection, and NA responses were determined by 60% plaque reduction assay. Similar to mock-immunized animals, i.m. immunization with UV-HRV14 did not induce detectable NA titers (**Figure 2**). In contrast, i.m. immunization with live HRV14 induced strong levels of NA titers at 3 weeks post-immunization, which were further increased in all animals after boosting (**Figure 2**, mean NA titer Log_2_ 9.3 ± 0.4 at 7 weeks). However, i.n. infection with HRV14 was less efficient at inducing NA response, with detectable levels of serum NA in only one out of five animals at 3 weeks p.i., and re-infection increased the number of animals with NA to a total of 3 out of 5.

### Immunization with HRV16 Induces Protection in Cotton Rats against HRV14 Challenge

**FIGURE 3:**
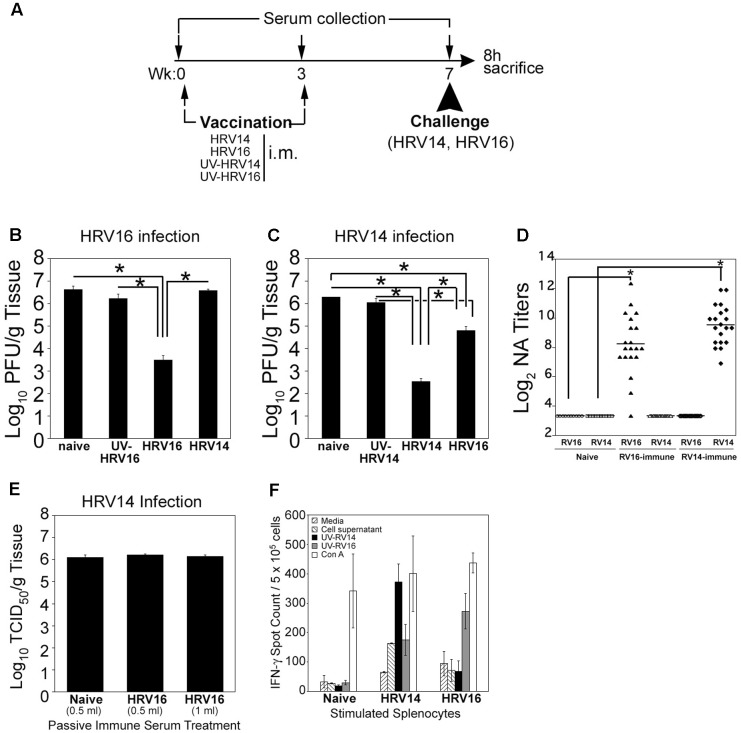
Homologous and heterologous HRV protection measured *in vivo*. **(A)** Scheme of immunization, serum collection, and challenge for this experiment. Animals were either unimmunized (naïve), immunized i.m. with UV- HRV14 or UV-HRV16, or immunized i.m. with live HRV14 or HRV16 (10^6^ PFU/100 μl). Three weeks after the initial immunization, animals were boosted with the same immunogen as indicated. At week 7, half of the animals in each group were challenged i.n. with HRV14 (10^6^ PFU/100 μl) and half with HRV16 (10^7^ PFU/100 μl), and all were euthanized 8 h later to determine lung viral titers. Lung viral titers following **(B)** HRV16 and **(C)** HRV14 infection are shown. *n* = 5 animals/group, data representative of two independent experiments. All data differences between different immunization groups with same infection were assessed by one-way ANOVA and individual differences were identified by Bonferroni *post hoc* test. **(D)** Homologous and heterologous serum NA titer of HRV14- or HRV16-immunized sera. NA titers were determined as described in the Materials and Methods section. The sera were assayed in duplicate and NA titer is expressed as Log_2_ geometric mean ± SEM, *n* = 10–20 sera/group. A NA titer of 3.3 ± 0.0 is the limit of detection of the assay. **(E)** Passive transfer of HRV16 hyper immune serum does not protect animals from HRV14 challenge. Animals were treated intraperitoneally with naïve serum (0.5 ml) or serum from animals immunized i.m. with HRV16 (0.5 or 1 ml). The following day, animals were challenged i.n. with 10^6^ PFU (100 μl/rat) of HRV14 and euthanized 8 h later to determine lung viral titers. *n* = 5 per group. **(F)** IFN-γ ELISPOT with splenocytes of naïve, HRV14-, and HRV16-immunized cotton rats after *in vitro* stimulation with control media, uninfected HeLa OH cell supernatant, homologous or heterologous UV-inactivated viruses, and Con A. Cotton rats were either unimmunized (naïve), immunized i.m. with live HRV14 or HRV16 (10^6^ PFU/100 μl). Three weeks after the initial immunization, animals were boosted with the same immunogen as indicated. At week 7, spleens were harvested and IFN-γ producing cells were estimated by ELISPOT assay as described in Section Materials and Methods. *n* = 3–5 rats/group.

We next studied the effect of HRV16 (a major group, A virus) and HRV14 (a major group, B virus) immunizations on the generation of either homologous or heterologous protective responses against either virus. We have demonstrated that i.m. immunization of cotton rats with live HRV14 or HRV16 serotypes generates strong NA responses against homologous virus (**Figure 2**; Blanco et al., 2014). Groups of naïve cotton rats, cotton rats immunized and boosted i.m. (day 0 and 3 weeks, respectively) with UV-HRV14 or UV-HRV16, and groups of cotton rats immunized and boosted i.m. with live HRV14 or HRV16, were used for i.n. homologous or heterologous virus challenge at 7 weeks after the first immunization. Five animals in each group were euthanized 8 h p.i. to determine lung viral titers (**Figure 3A**). Consistent with our previous data, animals vaccinated i.m. with live HRV16 virus were strongly protected against homologous HRV16 challenge, by reducing the lung viral titers by at least 3 Log_10_ (**Figure 3B**; Blanco et al., 2014). Importantly, live HRV14 i.m. immunization also strongly protected animals challenged with a homologous HRV14 infection, by reducing the lung viral titers by more than 3.5 Log_10_ (**Figure 3C**). Immunization with either UV-HRV16 or UV-HRV14 did not render protection to either HRV16 or HRV14 challenge, respectively (**Figures 3B,C**). When the generation of cross immunity was analyzed by cross-challenge experiments, cotton rats immunized i.m. with live HRV14 did not show heterologous protection against HRV16 challenge at the level of pulmonary viral replication (**Figure 3B**). However, i.m. vaccination with HRV16 partially protected animals against heterologous HRV14 challenge. Importantly, cotton rat vaccinated with live HRV16 i.m. and infected with HRV14 had ∼1.5 Log_10_ lower load of HRV14 in the lungs compared to naïve HRV14 infected animals, suggesting induction of detectable cross-protective immunity (**Figure 3C**).

Even though cross-protective immunity was observed *in vivo* in i.m. HRV16 immunized animals, no detectable NA titers against HRV14 were evident when serum samples from i.m. HRV16 immunized animals were tested *in vitro* against HRV14 (**Figure 3D**). This result was confirmed when passive transfer of serum containing high titer of NA against HRV16 to naïve animals did not impart protection against HRV14 challenge (**Figure 3E**). Furthermore, by ELISPOT assay, we observed increasing numbers of IFN-γ-producing splenocytes in animals immunized with HRV14 and HRV16 under homologous stimulation (UV-HRV14 or UV-HRV16, respectively), however no detectable increase of IFN-γ-producing splenocytes was detected under heterologous stimulation using this method (**Figure 3F**).

## Discussion

Our group has reported that the cotton rat is semi-permissive to major group HRV16 to a similar extent as the previously developed transgenic mice (Bartlett et al., 2008; Blanco et al., 2014). Here we show that cotton rats are susceptible to infection by another member of the major group, HRV14, with detectable replication in the URT and lower respiratory tract (LRT). The replication cycle of HRV14 is short-lived in nose and lung tissues of cotton rats. However, infectious virus titers in nose and lung tissues were measureable until 24 h p.i., which permits evaluation of vaccine efficacy or immunization strategy. We recently reported that EV-D68, another Picornavirus biologically similar to HRVs, also exhibited a similar replication cycle in cotton rats (Oberste et al., 2004; Patel et al., 2016). In contrast, other human respiratory viruses, e.g., RSV, and to a lesser extent, influenza virus, show extended replication (1–4 days p.i.) in nose and lung tissues of cotton rats (Ottolini et al., 2005; Blanco et al., 2013; Boukhvalova and Blanco, 2013). We believe that this shorter replication cycle of HRV may represent a hallmark of Picornaviruses in the respiratory tract of cotton rats and may be the result of differential tropism, interferon susceptibility, and/or receptor availability for this group of viruses.

Previously, we have shown that HRV16 infection in the cotton rat reproduces some aspects of HRV-associated human respiratory tract disease, causing detectable inflammation in the trachea, lower airways, and lung parenchyma, with mucus production, and transient induction of IFN-stimulated genes (Blanco et al., 2014). HRV16 infection induced mild, however, significant alveolitis, bronchiolitis and perivasculitis over 1–3 days p.i. Compared to HRV16, HRV14 infection seems to result in milder inflammatory response, which was reflected by low levels of cellular infiltration and lung pathology (**Figure 1D**). This result is quite surprising, however, for HRV infection, there is no comparative information available in the literature on the level of inflammation or lung pathology generated by different serotypes.

Similar to HRV16, i.m. immunization with HRV14 also generated a strong homologous NA response (Blanco et al., 2014; **Figure 2**). Infection and re-infection with HRV14 also resulted in induction of moderate NA in some animals; however, the induced NA titers were widely variable within the group compared to the i.m. HRV14-immunized group. Previously, we observed that a single i.n. infection with HRV16 did not induce a detectable NA response (Blanco et al., 2014). Similarly, it was shown in the mouse models that two or more HRV infections were necessary to generate detectable NA (McLean et al., 2012). As demonstrated by the lack of immune response generated by UV-inactivated viruses, viral replication seems to be an important component in the generation of NA in cotton rats (Blanco et al., 2014; **Figure 2**). It is possible that HRV14 or HRV16 replicate better in the lymph node draining from i.m. site of inoculation than from those draining from the respiratory mucosa. This is in agreement with our observations with EV-D68 that replicates in draining lymph nodes near the site of i.m. injection (Patel et al., 2016), possibly explaining the reason of higher NA response by i.m. inoculation compared to i.n. infection.

NA are considered to be important in protection against HRV infection. Experimental and natural infections in humans induce NA, which provide some protection against re-infection with the homologous HRV serotype (Barclay et al., 1989; Alper et al., 1996). Inactivated virus vaccinations given i.n. or i.m. in humans similarly induced NA and provided protection against disease caused by the homologous HRV serotype (Doggett et al., 1963; Mitchison, 1965; Perkins et al., 1969; Buscho et al., 1972; Hamory et al., 1975). We show here that live HRV14 or HRV16, used as immunogen and administered i.m., induce high levels of protective homologous NA that correlated with the ability of each of these viruses to undergo detectable replication in the nose and lung of i.n. infected cotton rats (Blanco et al., 2014). However, cotton rats immunized with minor group serotype HRV1B (that use LDL as a receptor) following identical vaccination schedule, neither generated NA nor replicated efficiently in the URT or LRT of these animals (Blanco et al., 2014). Additionally, HRV1B-immunized animals failed to render any protection against heterologous major group HRV16 challenge (Blanco et al., 2014). Taken together, these data suggest, first, that ICAM-1 in the cotton rat might harbor conserved features of the human counterpart that permits major group HRV attachment and entry. Second, our data supports the conclusion that viruses that generate strong humoral responses in cotton rats upon i.m. vaccination would potentially undergo productive replication in the airways. These two characteristics provide the basis for further development of an animal model for major group HRVs to test the probable vaccine candidates.

Development of a vaccine formulation and vaccination strategy against HRV infection has been cumbersome due to the antigenic diversity of this group of viruses (Glanville and Johnston, 2015). It is clear that a vaccine that targets this group of viruses will need to elicit an immune response that is broad enough to protect against a wide range of HRV serotypes. In a key study carried out more than 30 years ago using sera from rabbit and guinea pigs immunized with different HRV types, Cooney and collaborators defined several cross-neutralization relationships between HRV A and B types that allowed classification of different types into cross-reactivity groups (Cooney et al., 1975, 1982). More recently, genetic clustering of all human serotype strains has been performed based on the genomic sequence of the VP4/VP2 region (Savolainen et al., 2002) and based on the whole viral genome (Palmenberg et al., 2009). A report showed that immunization with recombinant HRV14 and HRV89 (two distantly related HRV strains) VP1 in mice and rabbits induced strong cross-NA response that showed cross-protection against distantly related HRV strains. However, the cross-protection was evidenced only by *in vitro* neutralization assays, and was based on the previously published serotype relationships (Edlmayr et al., 2011). In fact, there is limited *in vivo* data available to prove cross-protection or neutralization between different HRVs. Our data shows that the cotton rat can replicate two HRVs, both of which use ICAM-1 to gain entry into cells and major group A HRV16-immunized cotton rats were partially protected against major group B HRV14 challenge, indicating generation of cross-serotype immunity. Thus, our results indicated that the cotton rat model could be useful to measure the efficacy of vaccines against homologous, but also could be useful for dissecting heterologous protection once more serotypes of the major group are validated in the model.

To decipher the mechanisms underlying heterologous cross-protection, we have explored basic mechanisms of protection. First, using *in vitro* neutralization assays, we were unable to detect cross-NA response (**Figure 3D**). Secondly, we performed prophylactic treatment of naïve cotton rats using a hyperimmune pool of anti-HRV16 serum (Blanco et al., 2014). Although significant levels of NA were detected in treated animals, these animals failed to exhibit cross-protection upon HRV14 challenge (**Figure 3E**). Finally, to detect T-cell responses that could result in the cross-protection seen *in vivo*, we performed cotton rat-specific IFN-γ ELISPOT assays using splenocytes derived from HRV16-immunized animals and stimulated with control cell supernatant, UV-HRV14 or UV-HRV16 *in vitro*. We did not observe any difference in the number of IFN-γ-producing cells detected after stimulation with UV-HRV14 or control stimulation in the conditions and times assayed (**Figure 3F**). Overall, these additional experiments preclude us from concluding that either humoral or cellular responses are involved in cross-protection; however, they lay the groundwork for us to carry out more focused investigations further. Airway lymphocyte responses (using bronchoalveolar lavage, airway-draining lymph nodes, lung homogenates as source of lymphocytes) or cross-protective antibodies against HRV14, that are of mucosal in nature and are not neutralizing *in vitro*, could be sufficient to mediate neutralization *in vivo* or facilitate antibody-dependent cell-mediated cytotoxicity (ADCC) when present in the right quantity and distribution at the site of infection (Glanville et al., 2013). In addition, ELISPOT assay that measure IL-4- or IL-5- producing cells could also give some indication of type of response generated during cross-protection. It has been reported using a C57BL/6 model of HRV infection that immunization with recombinant VP0 protein from major group HRV16, combined with Th1 promoting adjuvants, induced antigen-specific Th1 responses in the airways, enhanced NA responses upon infection with minor group virus HRV1B and 29 and major group HRV14, and caused a rapid decrease in lung virus load in mice challenged with HRV1B, indicating that immunization with the capsid protein could prime animals to develop broader cross-reactive immunity against HRVs (McLean et al., 2012; Glanville et al., 2013). Importantly, Glanville et al. have shown that splenocytes from HRV16 VP0 protein immunized mice produced both IFN-γ and IL-5 when stimulated with VP0 peptides of HRV14. The lack of cross-stimulation of IFN-γ in splenocytes of HRV16 immunized cotton rats when stimulated with UV-HRV14 (**Figure 3F**) is likely the result of immunization with whole virus instead of a highly conserved peptide. A more recent report has shown that serum NA against many HRVs can be induced by polyvalent, inactivated HRVs plus alum as the adjuvant. Using formulations up to 25-valent in mice and 50-valent in rhesus macaques, Lee et al. showed that the extent of HRV vaccine immunogenicity was related to the quantity of input antigens, and valency was not a major factor affecting potency or breadth of the response. In addition, it was shown that the NA responses were type-specific and not cross-neutralizing because there was minimal *in vitro* neutralization activity induced by the 25-valent vaccine against 10 non-vaccine types (Lee et al., 2016).

In addition, we failed to document a correlation between immunization-mediated reduction in viral replication and the reduction of lung pathology to establish a clinically relevant protection. As inflammation due to primary HRV14 infection was mild, in the presented immunization experiment, a correlation between viral replication and disease could not been drawn. However, this result is not surprising since, as is observed in humans, the cotton rat model also fails to show benefit in clinical outcome when therapeutic administration of antibodies during RSV infection was used, despite complete protection of the lung from viral replication (Rodriguez et al., 1997; Malley et al., 1998; Prince et al., 2000). Although we could demonstrate partial heterologous protection by HRV16 immunization against HRV14 challenge, a reciprocal immunological relation between these two viruses could not be demonstrated (**Figures 3B,C**). It is entirely possible that an immunodominant epitope on HRV16 represents a minor epitope on HRV14, allowing for partial protection of animals vaccinated with HRV16, but not *vice versa*. Consistent with this hypothesis is the possibility that a distinct immunodominant epitope on HRV14 not present on HRV16 elicits homologous protection but not heterologous protection in HRV16-challenged animals.

Overall, our results are a step toward understanding the extent and strength of immunological cross-relatedness among HRVs *in vivo*. The capacity of this model to provide new insights in the development of a multivalent HRV vaccine will require the incorporation of additional serotypes of the major group into the analysis and to decipher the underlying mechanisms involved in the heterotypic protection.

## Author Contributions

MCP, JCGB, and AEK conceived and designed the experiments, wrote the paper. MCP, LMP, and AEK carried out the experiment, analyzed data. MSB and SNV contributed to technical and scientific discussions.

## Conflict of Interest Statement

The authors declare that the research was conducted in the absence of any commercial or financial relationships that could be construed as a potential conflict of interest.
